# Longitudinal evolution of sleep disturbances in early multiple system atrophy: a 2‐year prospective cohort study

**DOI:** 10.1186/s12916-023-03176-z

**Published:** 2023-11-22

**Authors:** Lingyu Zhang, Yanbing Hou, Chunyu Li, Qianqian Wei, Ruwei Ou, Kuncheng Liu, Junyu Lin, Tianmi Yang, Yi Xiao, Qirui Jiang, Bi Zhao, Huifang Shang

**Affiliations:** 1 Department of Neurology, Laboratory of Neurodegenerative Disorders, Rare Diseases Center, West China Hospital, Sichuan University, Chengdu, 610041 Sichuan China; 2 Health Management Center, General Practice Medical Center, West China Hospital, Sichuan University, Chengdu, Sichuan 610041 China

**Keywords:** Multiple system atrophy, REM sleep behavior disorder, Excessive daytime sleepiness, Parkinson’s disease-related sleep problems, Sleep disturbances

## Abstract

**Background:**

The progression of sleep disturbances remains unclear in patients with early multiple system atrophy (MSA). We aimed to explore the frequency, severity, and coexistence of 2-year longitudinal changes of sleep disturbances including REM sleep behavior disorder (RBD), excessive daytime sleepiness (EDS), and Parkinson’s disease-related sleep problems (PD-SP) in early MSA.

**Methods:**

MSA patients with a disease duration < 3 years were enrolled to complete a 2-year follow-up visit. Sleep disturbances including RBD, EDS, and PD-SP were assessed using the RBD Screening Questionnaire, Epworth sleepiness scale, and PD sleep scale-2, respectively.

**Results:**

A total of 220 patients with MSA enrolled in the study and 90 patients completed the 2-year follow-up visit. The score of all three sleep disturbances significantly increased over the 2-year follow-up in MSA and MSA with the predominant parkinsonism group (all *p* < 0.05). The frequency of PD-SP (from 14.5 to 26.7%) and EDS (from 17.7 to 37.8%) was progressively increased (all *p* < 0.05) except for RBD (from 51.8 to 65.6%, *p* = 0.152) over the 2-year follow-up in MSA. The frequency of coexistence of two or three sleep disturbances also increased over time. The most common sleep disturbance was RBD, followed by EDS and PD-SP over the 2-year follow-up.

**Conclusions:**

The present study demonstrated that the frequency of different types of sleep disturbances progressively increased except for RBD and the coexistence of two or three sleep disturbances became more common over time in early MSA. Our study suggested that the assessment and management of sleep disturbances should begin early in MSA.

**Supplementary Information:**

The online version contains supplementary material available at 10.1186/s12916-023-03176-z.

## Background

Multiple system atrophy (MSA) is a rare, adult-onset, progressive neurodegenerative disorder associated with a broad range of motor and non-motor symptoms (NMS) [1]. A growing number of studies confirmed that both motor symptoms and NMS of patients with MSA progress rapidly [2–4], leading to a shortened survival period [5–8] and a heavy burden of care on families.

A broad range of sleep disturbances are observed in patients with MSA and can occur in the early stage of MSA and indeed may precede the onset of motor symptoms [9–13]. A recent study reported that REM sleep behavior disorder (RBD) predated (pre-RBD) the disease onset in 27% of patients with MSA and the risk of death was higher in patients with pre-RBD [6]. RBD was present in nearly 50% of the patients with MSA [9, 14] or more than half [12], and the behaviors of RBD can be injurious to patients as well as bed partners. However, a few studies have focused on excessive daytime sleepiness (EDS) in MSA. A Spanish study that enrolled 86 patients with MSA reported that nearly one-third of patients with MSA experienced EDS [15]. Meanwhile, EDS was identified in 24% of the Japanese patients with MSA [13]. Additionally, our previous cross-sectional study found the frequency of RBD, EDS, and Parkinson’s disease (PD)-related sleep problems (PD-SP) defined using the PD sleep scale (PDSS-2) in patients with MSA was 49.7%, 27.3%, and 18.8%, respectively [9]. The frequency of coexistence of all three sleep-related symptoms was 7.3% [9]. Besides, sleep-related disorders were associated with the severity of disease in MSA [9].

Recently, a Spanish study found that the Epworth sleepiness scale (ESS) scores were significantly increased in patients with MSA at 2-year follow-up, while RBD Screening Questionnaire (RBD-SQ) scores were significantly decreased. However, the sample size is small with only 42 patients with MSA completing the 2-year follow-up, and the evolution of the frequency of sleep disturbances in MSA remains unknown. Consequently, there are limited data from prospective studies on the evolution of sleep disturbances in early MSA.

The present study aimed to explore the frequency, severity, and coexistence of 2-year longitudinal changes of sleep disturbances including RBD, EDS, and PD-SP in a large cohort of patients with early MSA.

## Methods

### Study design and population

We recruited patients with MSA from January 2018 to May 2022, referred from the Department of Neurology, West China Hospital, Sichuan University in the prospective cohort study. The disease duration of patients with MSA enrolled in the present study should be less than 3 years at baseline. Patients were excluded if the spinal cerebellar ataxia (SCA) genetic tests (SCA1, 2, 3, 6, 7), brain magnetic resonance imaging scan, or blood tests indicated other neurological disorders.

Patients underwent regular face-to-face interviews by neurologists at baseline, 1-year, and 2-year follow-ups until May 2023. A total of 231 patients were enrolled at baseline, seven patients voluntarily withdrew from the study, and four patients diagnosed with PD during the 1-year follow-up were excluded from the analysis. Finally, 220 patients completed the 1-year follow-up evaluation. Seventy-one patients could not come to the hospital due to death (*n* = 12), COVID-19 infection (*n* = 31), or wheelchair confinement (*n* = 28); 8 patients lost contact; 10 patients voluntarily withdrew from the study; 41 patients have not reached the time to completed the 2-year follow-up visit. Eventually, ninety patients completed the 2-year follow-up visit. All patients met the 2008 criteria for a probable diagnosis of MSA [16].

### Evaluation protocol

According to the predominant motor symptoms, patients were divided into two subtypes: MSA with predominant parkinsonism (MSA-P) or MSA with predominant cerebellar ataxia (MSA-C). Demographic and clinical data, including age, sex, age of onset, disease duration, and treatment, were recorded. The total daily Levodopa equivalent daily dose (LEDD) was calculated using the established methods [17]. Disease onset was defined as the initial presentation of any motor symptoms (Parkinsonism or cerebellar dysfunction) or autonomic features, except erectile dysfunction [16]. Disease duration was defined as the time between disease onset and evaluation.

The sleep disturbances evaluation included RBD, EDS, and PD-SP. The RBD-SQ, comprising a 10-item patient self-rating questionnaire covering the clinical features of RBD, is widely used as a screening tool for RBD [18]. RBD-SQ score ≥ 5 was defined as probable RBD. ESS is a simple, self-administered questionnaire for measuring daytime sleepiness [19], with a score of ≥ 10 indicating EDS. PD-SP was assessed using the PDSS-2 [20], which contains 15 individual items and was divided into three domains including disturbed sleep, motor symptoms at night, and PD symptoms at night. PD-SP was defined as a PDSS-2 score of ≥ 18. Disease severity was evaluated using the Unified Multiple System Atrophy Rating Scale (UMSARS) with part I (activities of daily living), part II (motor examination), part III (autonomic examination), and part IV (global disability) [21], and the total UMSARS score was calculated as the sum of parts I and II. The blood pressure measurements were taken at 1-, 3-, 5-, and 10-min intervals in the upright position, and were compared with the measurements in the supine position [22]. Orthostatic hypotension (OH) was defined as a reduction in the systolic blood pressure by at least 20 mmHg and/or diastolic blood pressure by at least 10 mmHg. Cognitive function was evaluated using a comprehensive and standardized cognitive screening tool (Montreal Cognitive Assessment (MoCA)) [23].

### Statistical analysis

All continuous data are presented as mean and standard deviation, and categorical variables as counts (percentages). Mann–Whitney *U* test was used to compare the continuous variables between different groups, since most of the data were not normally distributed. Chi-squared test was used to compare the categorical variables between different groups. When comparing the severity or frequency of the three types of sleep disturbances between patients with MSA-P and MSA-C, the logistic regression analysis was used to correct for confounding factors (age and LEDD) [24]. The 2-year change in the PDSS-2 score $=\frac{(\mathrm{PDSS}-2\mathrm{\ score\ at }2-\mathrm{year\ follow\ up }-\mathrm{ baseline\ PDSS}-2\mathrm{\ score})}{\left(\mathrm{date\ follow}-\mathrm{up\ in\ months}-\mathrm{date\ baseline\ in\ months}\right)/12}$, represents the progression of PD-SP. The 2-year change in the ESS score $=\frac{(\mathrm{ESS\ score\ at\ }2-\mathrm{year\ follow\ up }-\mathrm{\ baseline\ ESS\ score})}{\left(\mathrm{date\ follow}-\mathrm{up\ in\ months}-\mathrm{date\ baseline\ in\ months}\right)/12}$, represents the progression of EDS. The 2-year change in the RBDSQ score $=\frac{(\mathrm{RBDSQ\ score\ at }2-\mathrm{year\ follow\ up }-\mathrm{ baseline\ RBDSQ\ score})}{\left(\mathrm{date\ follow}-\mathrm{up\ in\ months}-\mathrm{date\ baseline\ in\ months}\right)/12}$. A generalized estimating equation (GEE) model with an exchangeable working correlation structure was used to investigate significant longitudinal sleep disturbance changes (score and frequency) in MSA after adjusting for age and LEDD, in this longitudinal observational study [4]. The Bonferroni correction was used for multiple comparisons. Additionally, we used GEE with multiple linear regression analysis to explore the factors associated with the score of PDSS-2, ESS, and RBDSQ. The models included all patients in the cohort and allowed for an association between repeated measurements acquired in the same patients. The independent variables included the following repeated measures: age, sex (male = 1, female = 0), diagnosis subtype (MSA-P = 1, MSA-C = 0), UMSARS total score, OH, and MoCA score. All data analyses were performed using the IBM SPSS Statistics software (version 26.0). *P* value < 0.05 was considered statistically significant.

## Results

### The clinical and demographic features of patients with MSA

In the current study, we recruited 231 patients with probable MSA at baseline and 220 patients (51.8% male, 48.6% MSA-P) were enrolled in analysis. Among the total of 220 patients with MSA, all completed 1-year follow-up assessment, and 90 patients completed the 2-year follow-up visit. There were no significant differences in the baseline clinical and demographic features between patients with MSA who were lost to follow-up and those who were not except for disease duration (Additional file 1: Table S1). The mean age of patients with MSA was 59.32 ± 8.09 years, and the mean disease duration was 1.68 ± 0.75 years at baseline. The score of PDSS-2 (11.13 ± 6.52 vs. 8.14 ± 5.27, *p* < 0.001) and ESS (6.13 ± 5.06 vs. 3.57 ± 3.87, *p* < 0.001) was higher in patients with MSA-P than in those with MSA-C after adjusting for age and LEDD. The frequency of PD-SP (20.6% vs. 8.8%, *p* = 0.016) and EDS (25.2% vs. 10.6%, *p* = 0.006) was also higher in patients with MSA-P than in those with MSA-C after adjusting for age and LEDD. Additionally, the score of RBDSQ and the frequency of RBD were not significantly different between patients with MSA-P and MSA-C. The score and frequency of sleep disturbances were not significantly different between male and female patients with MSA. The 2-year change in the PDSS-2, ESS, or RBDSQ score was not significantly different between MSA-P and MSA-C (all *p* > 0.05). The 2-year change in the ESS score was higher in male MSA than in female (*p* = 0.025), while the 2-year change in the PDSS-2 or RBDSQ score was not significantly different between male and female patients (all p > 0.05) (Table 1).

**Table 1:** The clinical and demographic features of patients with MSA

Variables	Time point	MSA	MSA-P	MSA-C	*p*-value	Male MSA	Female MSA	*p*-value
Number		220	107	113	-	114	106	-
Age (years)	Baseline	59.32 ± 8.09	60.73 ± 8.20	57.97 ± 7.78	0.022*	58.96 ± 8.75	59.71 ± 7.33	0.312
Sex (male, %)	Baseline	114, 51.8%	55, 51.4%	59, 52.2%	0.904	114, 100%	0, 0%	-
Diagnosis subtype (MSA-P, %)	Baseline	107, 48.6%	107, 100%	0, 0%	-	55, 48.2%	52, 49.1%	0.904
Age of onset (years)	Baseline	57.64 ± 8.09	59.06 ± 8.23	56.29 ± 7.74	0.021*	57.40 ± 8.73	57.90 ± 7.36	0.466
Disease duration (years)	Baseline	1.68 ± 0.75	1.67 ± 0.78	1.68 ± 0.73	0.814	1.55 ± 0.74	1.81 ± 0.75	0.016*
UMSARS-I score	Baseline	12.71 ± 5.79	12.94 ± 5.29	12.50 ± 6.25	0.423	12.27 ± 5.65	13.19 ± 5.93	0.154
	1-year FU	18.05 ± 7.10	17.21 ± 7.25	18.86 ± 6.90	0.086	17.63 ± 7.22	18.51 ± 6.98	0.402
	2-year FU	23.60 ± 7.47^(*n*=90)^	23.39 ± 8.53^(*n*=46)^	23.82 ± 6.27^(*n*=44)^	0.531	23.82 ± 8.40^(*n*=44)^	23.39 ± 6.55^(*n*=46)^	0.994
UMSARS-II score	Baseline	15.20 ± 5.87	15.64 ± 5.67	14.79 ± 6.04	0.199	14.57 ± 5.32	15.89 ± 6.35	0.100
	1-year FU	21.57 ± 7.10	20.43 ± 7.32	22.65 ± 6.75	0.020*	20.66 ± 6.88	22.56 ± 7.24	0.069
	2-year FU	26.66 ± 7.49^(*n*=90)^	27.33 ± 8.14^(*n*=46)^	25.95 ± 6.78^(*n*=44)^	0.645	26.93 ± 7.97^(*n*=44)^	26.39 ± 7.08^(*n*=46)^	0.923
UMSARS-IV score	Baseline	1.67 ± 0.74	1.67 ± 0.74	1.67 ± 0.74	0.974	1.50 ± 0.61	1.86 ± 0.81	< 0.001*
	1-year FU	2.48 ± 0.91	2.36 ± 0.88	2.59 ± 0.92	0.079	2.27 ± 0.86	2.70 ± 0.92	< 0.001*
	2-year FU	3.02 ± 1.02^(*n*=90)^	3.04 ± 1.10^(*n*=46)^	3.00 ± 0.94^(*n*=44)^	0.986	2.84 ± 1.03^(*n*=44)^	3.20 ± 0.98^(*n*=46)^	0.062
UMSARS total score	Baseline	27.92 ± 10.81	28.59 ± 10.03	27.28 ± 11.50	0.232	26.84 ± 10.05	29.08 ± 11.50	0.120
	1-year FU	39.63 ± 13.51	37.64 ± 13.87	41.51 ± 12.94	0.033*	38.29 ± 13.41	41.07 ± 13.53	0.151
	2-year FU	50.26 ± 14.39^(*n*=90)^	50.72 ± 16.22^(*n*=46)^	49.77 ± 12.37^(*n*=44)^	0.961	50.75 ± 15.84^(*n*=44)^	49.78 ± 13.02^(*n*=46)^	0.865
OH (%)	Baseline	83, 37.7%	34, 31.8%	49, 43.4%	0.076	44, 38.6%	39, 36.8%	0.783
	1-year FU	108, 49.1%	46, 43.0%	62, 54.9%	0.078	53, 46.5%	55, 51.9%	0.424
	2-year FU	51, 56.7%^(*n*=90)^	28, 60.9%^(*n*=46)^	23, 52.3%^(*n*=44)^	0.411	26, 59.1%^(*n*=44)^	25, 54.3%^(*n*=46)^	0.650
MoCA score	Baseline	23.90 ± 3.25^(*n*=211)^	23.99 ± 3.35^(*n*=100)^	23.82 ± 3.17^(*n*=111)^	0.678	24.30 ± 3.31^(*n*=113)^	23.44 ± 3.14^(*n*=98)^	0.052
	1-year FU	22.09 ± 3.63^(*n*=211)^	22.43 ± 3.73^(*n*=100)^	21.79 ± 3.52^(*n*=111)^	0.192	22.41 ± 3.64^(*n*=113)^	21.73 ± 3.60^(*n*=98)^	0.196
	2-year FU	21.09 ± 4.32^(*n*=78)^	21.84 ± 4.11^(*n*=37)^	20.41 ± 4.44^(*n*=41)^	0.166	21.53 ± 4.28^(*n*=38)^	20.68 ± 4.37^(*n*=40)^	0.272
PDSS-2 score	Baseline	9.60 ± 6.08	11.13 ± 6.52	8.14 ± 5.27	< 0.001^#^	8.81 ± 5.63	10.44 ± 6.46	0.064
ESS score	Baseline	4.81 ± 4.66	6.13 ± 5.06	3.57 ± 3.87	< 0.001^#^	5.10 ± 4.78	4.51 ± 4.53	0.359
RBDSQ score	Baseline	5.04 ± 3.40	4.92 ± 3.11	5.15 ± 3.66	0.917	4.96 ± 3.40	5.11 ± 3.40	0.711
2-year change in the PDSS-2 score	-	1.23 ± 3.26^(*n*=90)^	1.24 ± 3.94^(*n*=46)^	1.22 ± 2.41^(*n*=44)^	0.977	0.63 ± 2.61^(*n*=44)^	1.80 ± 3.73^(*n*=46)^	0.051
2-year change in the ESS score	-	1.63 ± 2.57^(*n*=90)^	1.72 ± 2.78^(*n*=46)^	1.52 ± 2.35^(*n*=44)^	0.865	2.32 ± 2.75^(*n*=44)^	0.96 ± 2.21^(*n*=46)^	0.025*
2-year change in the RBDSQ score	-	0.28 ± 1.76^(*n*=90)^	0.52 ± 1.60^(*n*=46)^	0.02 ± 1.89^(*n*=44)^	0.429	0.26 ± 1.83^(*n*=44)^	0.30 ± 1.70^(*n*=46)^	0.958
Presence of PD-SP (*n*, %)	Baseline	32, 14.5%	22, 20.6%	10, 8.8%	0.016^#^	13, 11.4%	19, 17.9%	0.170
Presence of EDS (*n*, %)	Baseline	39, 17.7%	27, 25.2%	12, 10.6%	0.006^#^	24, 21.1%	15, 14.2%	0.180
Presence of RBD (*n*, %)	Baseline	114, 51.8%	55, 51.4%	59, 52.2%	0.904	57, 50.0%	57, 53.8%	0.576
LEDD (mg/d)	Baseline	168.33 ± 236.76	252.53 ± 271.58	88.61 ± 163.15	< 0.001*	188.69 ± 238.31	146.44 ± 234.23	0.073
Use of sleep-related medications (*n*, %)	Baseline	16, 7.3%	9, 8.4%	7, 6.2%	0.527	9, 7.9%	7, 6.6%	0.713

*MSA* multiple system atrophy, *MSA-P* multiple system atrophy with predominant parkinsonism, *MSA-C* multiple system atrophy with predominant cerebellar ataxia, *UMSARS* Unified Multiple System Atrophy Rating Scale, *OH* orthostatic hypotension, *MoCA* Montreal Cognitive Assessment, *PDSS-2* Parkinson’s disease sleep scale-2, *ESS* Epworth sleepiness scale, *RBDSQ* rapid eye movement sleep behavior disorder screening questionnaire, *PD-SP* Parkinson’s disease-related sleep problems, *EDS* excessive daytime sleepiness, *RBD* rapid eye movement sleep behavior disorder, *LEDD* levodopa equivalent daily doses, *FU* follow-up

^*^Significant difference

^#^Adjusting for age and LEDD

### Score of sleep disturbances over the 2-year follow-up

**Fig. 1:**
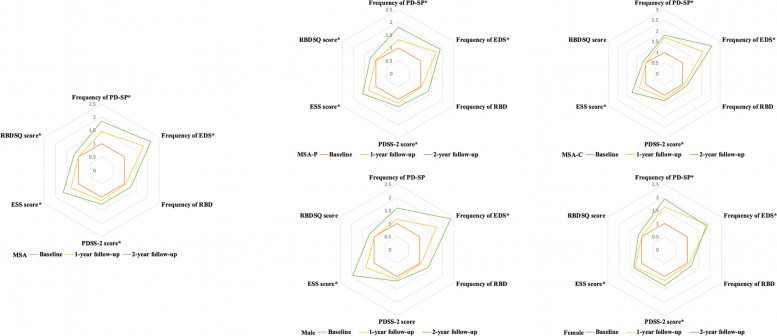
Comparison of the frequency and score of sleep disturbances over the 2-year follow-up in different subgroups. Illustrates: In the radar chart, all values were normalized to baseline values (mean follow-up score/mean baseline score, or follow-up frequency/baseline frequency), and baseline values were set to 1.0. * Significant difference MSA, multiple system atrophy; MSA-P, multiple system atrophy with predominant parkinsonism; MSA-C, multiple system atrophy with predominant cerebellar ataxia; PDSS-2, Parkinson’s disease sleep scale-2; ESS, Epworth sleepiness scale; RBDSQ, rapid eye movement sleep behavior disorder screening questionnaire; PD-SP, Parkinson’s disease-related sleep problems; EDS, excessive daytime sleepiness; RBD, rapid eye movement sleep behavior disorder

In the total MSA group, the score of PDSS-2, ESS, and RBDSQ was significantly increased over the 2-year follow-up (all *p* < 0.05), even after adjusting for age and LEDD. In the MSA-P group, the score of PDSS-2, ESS, and RBDSQ was significantly increased over the 2-year follow-up (all *p* < 0.05). After adjusting for age and LEDD, the score of ESS and RBDSQ remained significantly increased (all *p* < 0.05), while the score of PDSS-2 did not (*p* = 0.234). In the MSA-C group, the score of PDSS-2 and ESS was significantly increased over the 2-year follow-up (all *p* < 0.05), even after adjusting for age and LEDD. The score of RBDSQ was not significantly increased over the 2-year follow-up (all *p* > 0.05). In the male patients in the MSA group, the score of ESS was significantly increased over the 2-year follow-up (all *p* < 0.001), while the score of PDSS-2 and RBDSQ was not (all *p* > 0.05). In the female patients with MSA, the score of PDSS-2 and ESS was significantly increased over the 2-year follow-up (all *p* < 0.001), while the score of RBDSQ was not (*p* = 0.144) (Table 2 and Fig. 1).

**Table 2:** The comparison of the score and frequency of sleep disturbances at baseline and 1- and 2-year follow-ups

Time point	PDSS-2 score	*p*-value	*p*-value^#^	Post hoc tests^#^	ESS score	*p*-value	*p*-value^#^	Post hoc tests^#^	RBDSQ score	*p*-value	*p*-value^#^	Post hoc tests^#^
MSA												
Baseline	9.60 ± 6.08	< 0.001*	0.033*	-	4.81 ± 4.66	< 0.001*	< 0.001*	**a,b,c**	5.04 ± 3.40	0.010*	0.009*	**c**
1-year FU	10.97 ± 7.05				6.41 ± 5.96				5.04 ± 3.01			
2-year FU	12.36 ± 8.51				8.00 ± 6.93				5.97 ± 2.94			
MSA-P				α								
Baseline	11.13 ± 6.52	0.014*	0.234	-	6.13 ± 5.06	< 0.001*	0.001*	**a,b,c**	4.92 ± 3.11	0.004*	0.010*	**c**
1-year FU	12.64 ± 7.49				8.16 ± 6.31				4.99 ± 2.88			
2-year FU	14.35 ± 9.53				9.70 ± 7.42				6.11 ± 2.93			
MSA-C												
Baseline	8.14 ± 5.27	0.001*	0.026*	**b**	3.57 ± 3.87	< 0.001*	0.003*	**b**	5.15 ± 3.66	0.617	0.409	-
1-year FU	9.39 ± 6.23				4.76 ± 5.10				5.09 ± 3.15			
2-year FU	10.27 ± 6.80				6.23 ± 5.96				5.82 ± 2.98			
Male MSA												
Baseline	8.81 ± 5.63	0.057	0.480	-	5.10 ± 4.78	< 0.001*	< 0.001*	**a,b,c**	4.96 ± 3.40	0.070	0.098	-
1-year FU	9.82 ± 6.48				6.95 ± 6.43				4.94 ± 3.11			
2-year FU	10.48 ± 7.83				10.00 ± 7.29				6.02 ± 3.08			
Female MSA												
Baseline	10.44 ± 6.46	< 0.001*	0.021*	**b**	4.51 ± 4.53	< 0.001*	0.061	**-**	5.11 ± 3.40	0.144	0.089	-
1-year FU	12.22 ± 7.45				5.84 ± 5.38				5.15 ± 2.92			
2-year FU	14.15 ± 8.83				6.09 ± 6.06				5.91 ± 2.84			
Time point	Presence of PD-SP	*p*-value	*p*-value^#^	Post hoc tests^#^	Presence of EDS	*p*-value	*p*-value^#^	Post hoc tests^#^	Presence of RBD	*p*-value	*p*-value^#^	Post hoc tests^#^
MSA												
Baseline	32 (14.5%)	0.001*	0.108	-	39 (17.7%)	< 0.001*	< 0.001*	**a,b,c**	114 (51.8%)	0.152	0.093	-
1-year FU	46 (20.9%)				71 (32.3%)				121 (55.0%)			
2-year FU	24 (26.7%)				34 (37.8%)				59 (65.6%)			
MSA-P												
Baseline	22 (20.6%)	0.026*	0.232	-	27 (25.2%)	< 0.001*	< 0.001*	**a**	55 (51.4%)	0.063	0.162	-
1-year FU	29 (27.1%)				46 (43.0%)				58 (54.2%)			
2-year FU	17 (37.0%)				22 (47.8%)				32 (69.6%)			
MSA-C												
Baseline	10 (8.8%)	0.041*	0.160	-	12 (10.6%)	< 0.001*	0.002*	**a**	59 (52.2%)	0.783	0.209	-
1-year FU	17 (15.0%)				25 (22.1%)				63 (55.8%)			
2-year FU	7 (15.9%)				12 (27.3%)				27 (61.4%)			
Male MSA												
Baseline	13 (11.4%)	0.127	0.878	-	24 (21.1%)	< 0.001*	< 0.001*	**a,b,c**	57 (50.0%)	0.257	0.530	-
1-year FU	15 (13.2%)				42 (36.8%)				60 (52.6%)			
2-year FU	8 (18.2%)				22 (50.0%)				30 (68.2%)			
Female MSA												
Baseline	19 (17.9%)	0.003*	0.022*	**a**	15 (14.2%)	< 0.001*	0.002*	**a**	57 (53.8%)	0.546	0.080	-
1-year FU	31 (29.2%)				29 (27.4%)				61 (57.5%)			
2-year FU	16 (34.8%)				12 (26.1%)				29 (63.0%)			

*MSA* multiple system atrophy, *MSA-P* multiple system atrophy with predominant parkinsonism, *MSA-C* multiple system atrophy with predominant cerebellar ataxia, *PDSS-2* Parkinson’s disease sleep scale-2, *ESS* Epworth sleepiness scale, *RBDSQ* rapid eye movement sleep behavior disorder screening questionnaire, *PD-SP* Parkinson’s disease-related sleep problems, *EDS* excessive daytime sleepiness, *RBD* rapid eye movement sleep behavior disorder, *FU* follow-up

^*^Significant difference

^#^Adjusting for age and LEDD

Post hoc tests (Bonferroni correction):

Baseline vs 1-year follow-up:** a**, significant

Baseline vs 2-year follow-up:** b**, significant

1- vs 2-year follow-up:** c**, significant

The comparison of the score of the PDSS-2 domain at baseline and 1- and 2-year follow-ups was shown in Additional file 1: Table S2. We found that the score of disturbed sleep was significantly increased over the 2-year follow-up (all *p* < 0.05) in the total MSA, MSA-C, and male patients with MSA groups, even after adjusting for age and LEDD. However, after adjusting for age and LEDD, the score of motor symptoms at night and PD symptoms at night was not significantly increased over the 2-year follow-up in all groups (all *p* > 0.05).

### Frequency of sleep disturbances over the 2-year follow-up

In the total MSA group, the most common sleep disturbance was RBD, followed by EDS and PD-SP over the 2-year follow-up. The frequency of EDS was significantly increased from 17.7 to 37.8% over the 2-year follow-up (*p* < 0.001), even after adjusting for age and LEDD. The frequency of PD-SP was significantly increased from 14.5 to 26.7% over the 2-year follow-up (*p* = 0.001), while the finding did not remain after adjusting for age and LEDD (*p* = 0.108). In the MSA-P group, the frequency of EDS was significantly increased from 25.2 to 47.8% over the 2-year follow-up (*p* < 0.001), even after adjusting for age and LEDD. The frequency of PD-SP was significantly increased from 20.6 to 37.0% over the 2-year follow-up (*p* = 0.026), while the finding did not remain after adjusting for age and LEDD (*p* = 0.232). In the MSA-C group, the frequency of EDS significantly increased from 10.6 to 27.3% over the 2-year follow-up (all *p* < 0.05), even after adjusting for age and LEDD. The frequency of PD-SP was significantly increased from 8.8 to 15.9% over the 2-year follow-up (*p* = 0.041), while the finding did not remain after adjusting for age and LEDD (*p* = 0.160). In the male patients in the MSA group, the frequency of EDS was significantly increased from 21.1 to 50.0% over the 2-year follow-up (*p* < 0.001), even after adjusting for age and LEDD. The frequency of PD-SP was not significantly increased over the 2-year follow-up (all *p* > 0.05). In the female patients in the MSA group, the frequency of PD-SP (from 17.9 to 34.8%) and EDS (from 14.2 to 26.1%) was significantly increased over the 2-year follow-up (all *p* < 0.05), even after adjusting for age and LEDD. However, the frequency of RBD was not significantly increased over the 2-year follow-up in any group (all *p* > 0.05) (Table 2 and Fig. 1).

### Coexistence of sleep disturbances over the 2-year follow-up

**Fig. 2:**
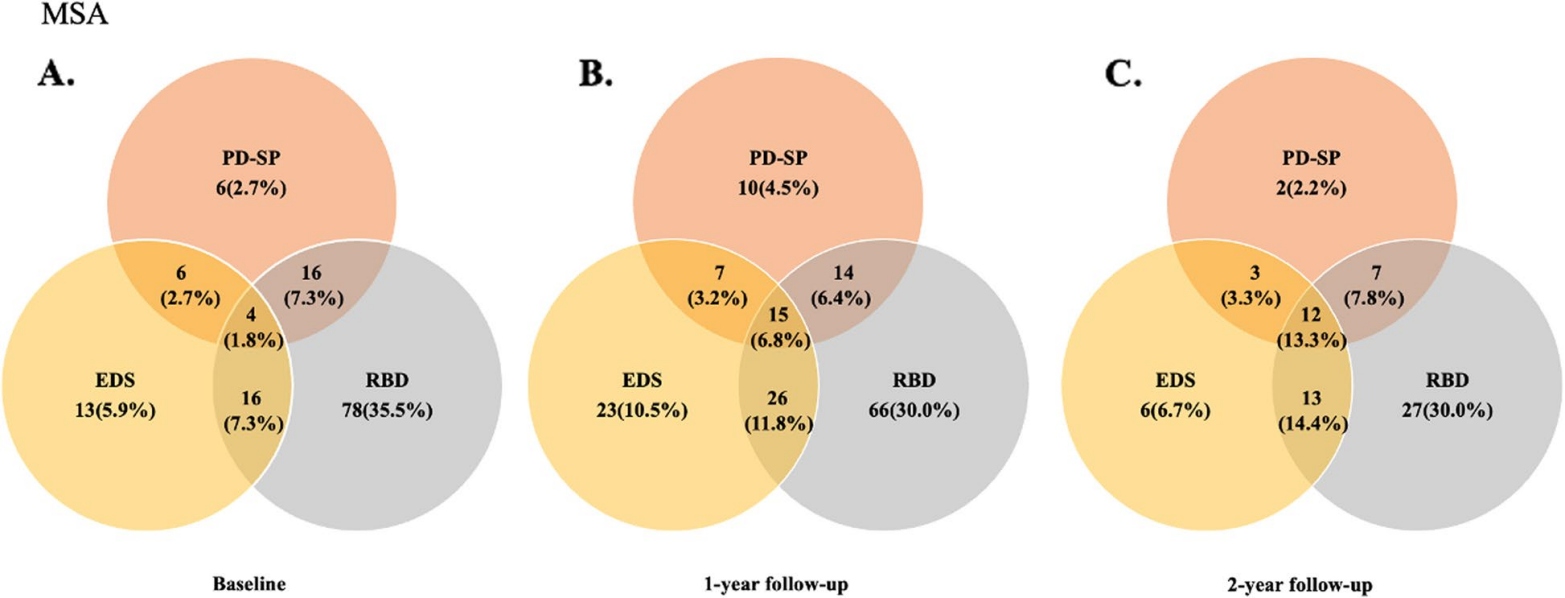
Frequency and overlap of sleep disturbances in patients with MSA at baseline (**A**) and 1- (**B**) and 2-year (**C**) follow-ups. MSA, multiple system atrophy; EDS, Excessive daytime sleepiness; PD-SP, Parkinson’s disease-related sleep problems; RBD, rapid eye movement sleep behavior disorder

In the total MSA group, the frequency of patients with MSA did not report any sleep disturbances at baseline, 1-year, and 2-year follow-up was 36.8%, 26.8%, and 22.2%, respectively. The frequency of overlap in two types of sleep disturbances at baseline, 1-year, and 2-year follow-up was 17.3%, 21.4%, and 25.5%, respectively. The frequency of coexistence of all three sleep disturbances at baseline, 1-year, and 2-year follow-up was 1.8%, 6.8%, and 13.3%, respectively (Fig. 2).

**Fig. 3:**
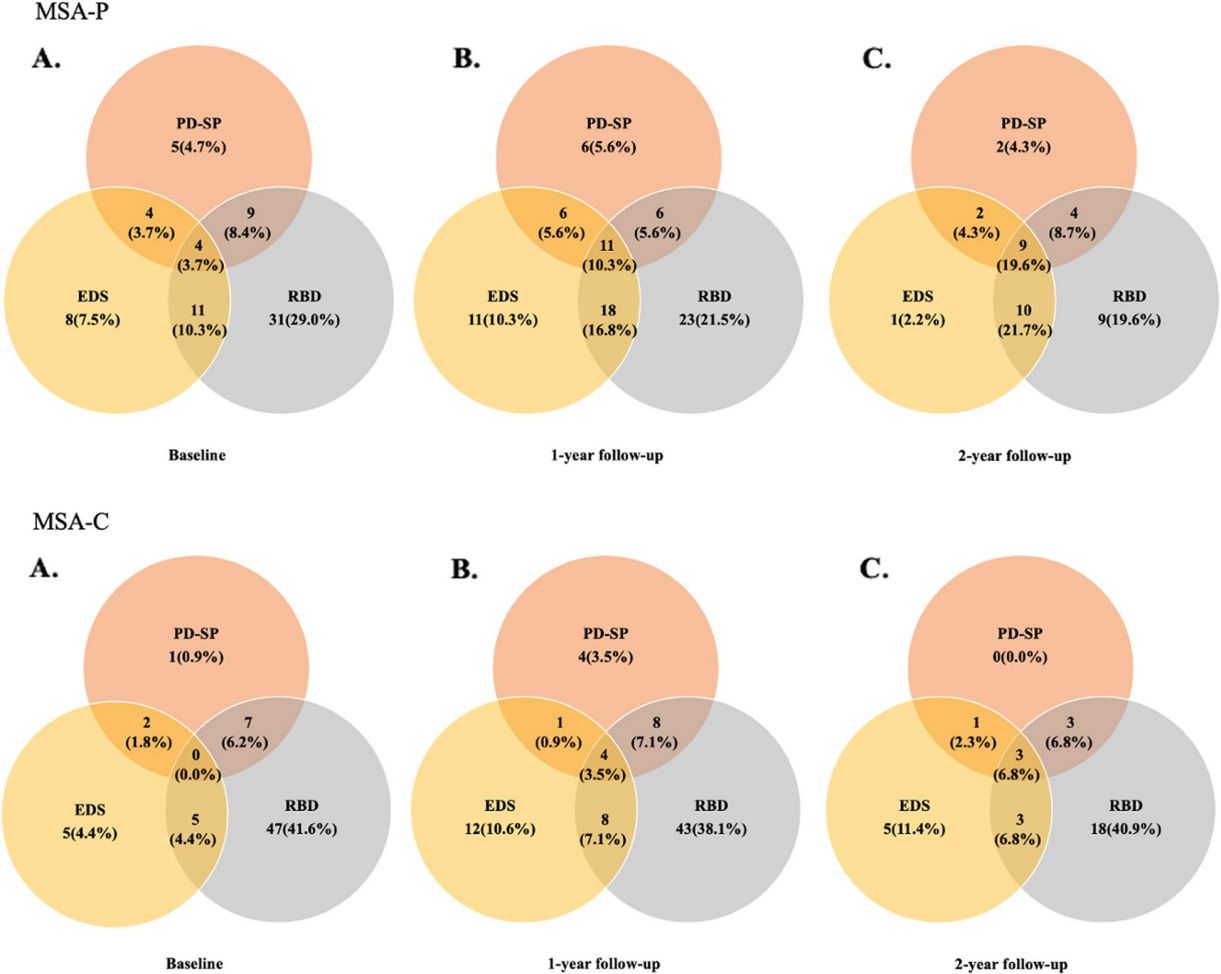
Frequency and overlap of sleep disturbances in patients with MSA-P and MSA-C at baseline (**A**) and 1- (**B**) and 2-year (**C**) follow-ups. MSA-P, multiple system atrophy with predominant parkinsonism; MSA-C, multiple system atrophy with predominant cerebellar ataxia; EDS, Excessive daytime sleepiness; PD-SP, Parkinson’s disease-related sleep problems; RBD, Rapid eye movement sleep behavior disorder

In the MSA-P group, the frequency of overlap in two types of sleep disturbances at baseline, 1-year, and 2-year follow-up was 22.4%, 28.0%, and 34.7%, respectively. The frequency of coexistence of all three sleep disturbances at baseline, 1-year, and 2-year follow-up was 3.7%, 10.3%, and 19.6%, respectively. In the MSA-C group, the frequency of overlap in two types of sleep disturbances at baseline, 1-year, and 2-year follow-up was 12.4%, 15.1%, and 15.9%, respectively. The frequency of coexistence of all three sleep disturbances at baseline, 1-year, and 2-year follow-up was 0.0%, 3.5%, and 6.8%, respectively (Fig. 3).

**Fig. 4:**
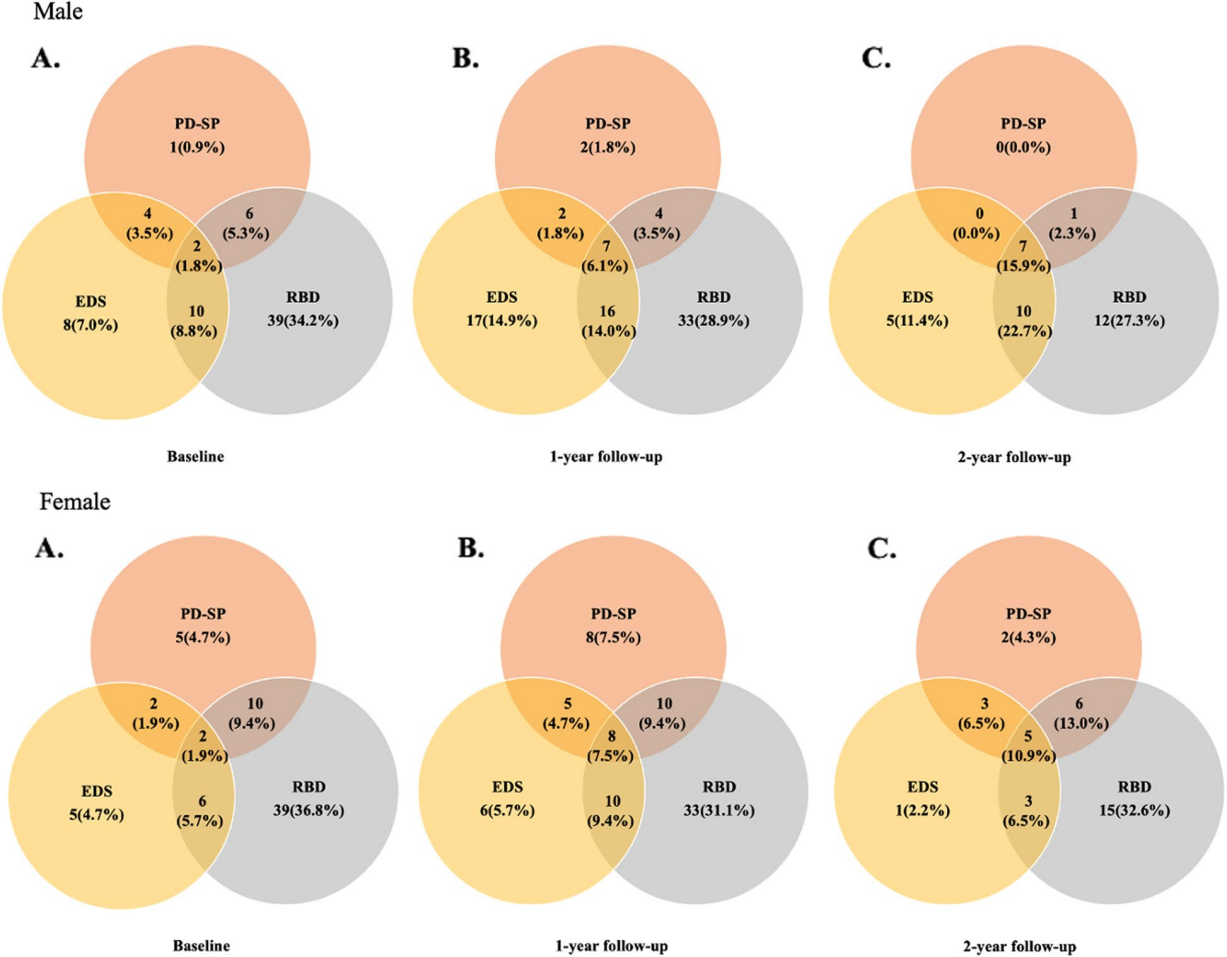
Frequency and overlap of sleep disturbances in male and female patients with MSA at baseline (**A**) and 1- (**B**) and 2-year (**C**) follow-ups. MSA, multiple system atrophy; EDS, excessive daytime sleepiness; PD-SP, Parkinson’s disease-related sleep problems; RBD, rapid eye movement sleep behavior disorder

In the male patients with MSA, the frequency of overlap in two types of sleep disturbances increased from 17.6 to 25.0% over the 2-year follow-up. The frequency of coexistence of all three sleep disturbances increased from 1.8 to 15.9% over the 2-year follow-up. In the female patients with MSA, the frequency of overlap in two types of sleep disturbances increased from 17.0 to 26.0% over the 2-year follow-up. The frequency of coexistence of all three sleep disturbances increased from 1.9 to 10.9% over the 2-year follow-up (Fig. 4).

### Factors associated with sleep disturbances in patients with MSA over the 2-year follow-up

The GEE model showed that the sex (*B* =  − 2.063, *p* = 0.006), diagnosis subtype (*B* = 3.146, *p* < 0.001), and UMSARS total score (*B* = 0.080, *p* < 0.001) were associated with the score of PDSS-2; that sex (*B* = 1.537, *p* = 0.012), diagnosis subtype (*B* = 3.066, *p* < 0.001), UMSARS total score (*B* = 0.085, *p* < 0.001), and MoCA score (*B* =  − 0.282, *p* < 0.001) were associated with the score of ESS; and that the UMSARS total score (*B* = 0.027, *p* = 0.014) were associated with the score of RBDSQ (Additional file 1: Table S3).

## Discussion

In the largest prospective cohort study, we assessed the baseline severity and frequency and longitudinal evolution of sleep disturbances in early MSA. Our data demonstrated that the severity and frequency of PD-SP and EDS were progressively increased. Coexistence of two or three sleep disturbances became more common over time. The most common sleep disturbance was RBD, followed by EDS and PD-SP over the 2-year follow-up.

The novel aspect of the current study is that we conducted the largest prospective cohort study to assess the longitudinal evolution of sleep disturbances in early MSA over 2 years. Our previous cross-sectional study has shown that the most common sleep disturbance in patients with MSA was RBD, followed by EDS and PD-SP [9]. Similarly, in the present study, we found that RBD was the most common sleep disturbance in early MSA, followed by EDS and then PD-SP, which remains consistent even over the 2-year follow-up. An American study that included 171 patients with autopsy-confirmed MSA in the Mayo Clinic brain bank showed that RBD was present in 48% of the patients with MSA [14], which was supported our results. Another study enrolled only 61 MSA with disease duration ≤ 3 years reported that RBD was present in 90.2% of the patients [25], which was higher than our study. This inconsistency may be attributed to the small sample size of the above study. It has been reported that nearly one-third of patients with MSA experienced EDS [13, 15], which was consistent with our results, as we found that EDS was present in 17.7% of the patients with early MSA at baseline and increased to 37.8% over the 2-year follow-up.

It is notable that the score of all three types of sleep disturbances significantly increased over the 2-year follow-up. Additionally, the domain score of disturbed sleep in PDSS-2 significantly increased over the 2-year follow-up, while the domain score of motor symptoms at night and PD symptoms at night were not. However, only one Spanish study on only 42 patients with MSA completed the 2-year follow-up and reported that the severity of EDS was significantly increased, while the score of RBDSQ significantly decreased over the 2-year follow-up [4]. The decrease of score of RBDSQ was owing to the treatment and underestimate of RBD as the disease progresses [4]. In our study, the frequency of use of sleep-related medications was low (ranging from 7.3% at baseline to 15.6% at 2-year follow-up). Thus, we consider that the results in the current study may better reflect the progression of sleep disturbances in MSA. Additionally, we found that the severity and frequency of PD-SP and EDS were more severe in patients with MSA-P than in MSA-C. And the MSA-P subtype was significantly associated with the scores of PD-SP and EDS. The disease severity was significantly associated with all sleep disturbances in MSA. These results were consistent with our previous study [9]. Another Chinese study also reported that the score of RBDSQ and frequency of RBD were not significantly different between patients with MSA-P and MSA-C [26], which supported our results. Additionally, we first found that the severity and frequency of sleep disturbances were not significantly different between male and female patients with MSA.

RBD is a sleep behavior disorder characterized by abnormal behaviors and loss of muscle atonia during rapid eye movement sleep, which is generally associated with synucleinopathies, such as PD and MSA [27]. The appearance of RBD may precede years before the onset of MSA [27, 28], representing a key prodromal marker. It has been demonstrated that the pre-RBD is associated with more rapid disease progression and survival in MSA [6]. Pathological changes to the lateral dorsal tegmentum (LDT)/pedunculopontine nucleus (PPN) and locus coeruleus (LC) in MSA may lead to RBD [29]. More than half of the patients with MSA experienced RBD in the early stage regardless of the subtype of MSA, indicating that the dysfunctions in the LDT/PPN and LC occurred early in MSA with different subtypes. EDS and PD-SP in patients with MSA are caused by a variety of factors, and there is some correlation between the two. The frequency of overlap of PD-SP and EDS remained low over the 2-year follow-up. These two sleep disturbances did not influence the majority of MSA. Sleep-disordered breathing and sleep efficiency may be associated with EDS in MSA [15], but the loss of dopaminergic neurons in the ventral periaqueductal gray matter may play a key role in contributing to EDS in MSA [30]. EDS seems to be related to different causes. The increase of severity and frequency of EDS over the 2-year follow-up may reflect the increasing loss of dopaminergic neurons in MSA with the disease progression. Additionally, we also considered the potential impact of dopamine replacement drugs on sleep disturbances. The progression of PD-SP and EDS was significant even after adjusting for LEDD and age, indicating the overall impact of LEDD on PD-SP and EDS was small.

RBD may indicate the emergence of synucleinopathies by decades, often preceding motor symptom onset by several years and representing a key prodromal marker of MSA [29, 31, 32]. However, less is known about the presence of PD-SP or EDS in patients with early stage of MSA. Our research results just filled this gap. Patients with MSA can develop PD-SP or EDS besides RBD in the early stage of MSA. These results suggest that MSA patients should be asked about the presence of sleep disturbances by neurologists even in the early stage of the disease. Those sleep disturbances can be considered as an optimal early diagnostic/prognostic maker for MSA. Our study suggested that the management of sleep disturbances should begin early in MSA.

The current study has several advantages. First, we enrolled a large number of early MSA to assess the longitudinal evolution of sleep disturbances over a 2-year follow-up. Second, we assessed not only the evolution of a single sleep disturbance but also the coexistence of different sleep disturbances using multiple sleep-related scales. Additionally, subgroup analysis was also conducted according to the subtypes and gender. Despite these advantages, there are some limitations that cannot be ignored. First, we did not perform an objective assessment of RBD, sleep-disordered breathing, or periodic limb movements using polysomnography. However, sleep-related scales are simple tools that can be used in routine clinical practice, since the polysomnography is not easily available. The second limitation was the lack of healthy controls. Third, there was a lack of postmortem pathological confirmation of MSA.

## Conclusions

As we all know, this large prospective cohort study showed that the severity and frequency of different types of sleep disturbances progressively increased except for the RBD over time in early MSA. The frequency of coexistence of two or three sleep disturbances also increased over time. Our study suggested that the assessment and management of sleep disturbances should begin early in MSA.

### Supplementary Information

**Additional file 1: Table S1.** Baseline clinical and demographic features of loss of FU and non-loss of FU patients with MSA. **Table S2.** The comparison of the score of PDSS-2 domain at baseline and 1- and 2-year follow-ups. **Table S3.** Factors associated with score of PDSS-2, ESS, and RBDSQ in patients with MSA.

## Data Availability

The datasets used and/or analyzed during the current study are available from the corresponding author on reasonable request.

## Abbreviations

EDS: Excessive daytime sleepiness
ESS: Epworth sleepiness scale
GEE: Generalized estimating equation
LC: Locus coeruleus
LDT: Lateral dorsal tegmentum
LEDD: Levodopa equivalent daily dose
MoCA: Montreal Cognitive Assessment
MSA: Multiple system atrophy
MSA-C: Multiple system atrophy with predominant cerebellar ataxia
MSA-P: Multiple system atrophy with predominant parkinsonism
NMS: Non-motor symptoms
OH: Orthostatic hypotension
PD: Parkinson’s disease
PD-SP: Parkinson’s disease-related sleep problems
PDSS-2: Parkinson’s disease sleep scale
PPN: Pedunculopontine nucleus
pre-RBD: REM sleep behavior disorder predated
RBD: REM sleep behavior disorder
RBD-SQ: REM sleep behavior disorder screening questionnaire
SCA: Spinal cerebellar ataxia
UMSARS: Unified Multiple System Atrophy Rating Scale
