# Supplementary material for: Longitudinal evolution of sleep disturbances in early multiple system atrophy: a 2‐year prospective cohort study
Source: BMC Med. 2023 Nov 22;21:454. doi: 10.1186/s12916-023-03176-z (PMC10664599; doi:10.1186/s12916-023-03176-z)
Supplement: Supplementary file 1 — Additional file 1: Table S1. Baseline clinical and demographic features of loss of FU and non-loss of FU patients with MSA. Table S2. The comparison of the score of PDSS-2 domain at baseline and 1- and 2-year follow-ups. Table S3. Factors associated with score of PDSS-2, ESS, and RBDSQ in patients with MSA. [file 12916_2023_3176_MOESM1_ESM.docx]

Table S1 Baseline clinical and demographic features of loss of FU and non-loss of FU patients with MSA

| variables | MSA | | |
| --- | --- | --- | --- |
|  | Non-loss of FU | Loss of FU | p value |
| Number | 90 | 130 | - |
| Age (years) | 58.71±8.10 | 59.73±8.09 | 0.247 |
| Sex (male, %) | 44, 48.9% | 70, 53.8% | 0.469 |
| Diagnosis subtype (MSA-P, %) | 46, 51.1% | 61, 46.9% | 0.541 |
| Age of onset (years) | 56.84±8.04 | 58.19±8.10 | 0.158 |
| Disease duration (years) | 1.87±0.78 | 1.54±0.71 | 0.004* |
| UMSARS-I score | 12.83±5.00 | 12.63±6.30 | 0.553 |
| UMSARS-II score | 14.77±5.36 | 15.51±6.19 | 0.495 |
| UMSARS-IV score | 1.67±0.69 | 1.68±0.77 | 0.853 |
| UMSARS total score | 27.60±9.48 | 28.14±11.67 | 0.941 |
| PDSS-2 score | 9.86±6.58 | 9.42±5.73 | 0.843 |
| ESS score | 4.62±4.98 | 4.95±4.43 | 0.309 |
| RBDSQ score | 5.34±3.44 | 4.82±3.36 | 0.249 |
| Presence of PD-SP (n, %) | 15, 16.7% | 17, 13.1% | 0.458 |
| Presence of EDS (n, %) | 15, 16.7% | 24, 18.5% | 0.732 |
| Presence of RBD (n, %) | 53, 58.9% | 61, 46.9% | 0.081 |
| LEDD (mg/d) | 168.73±233.29 | 168.06±240.03 | 0.947 |
| Use of sleep-related medications (n, %) | 8, 8.9% | 8, 6.2% | 0.442 |

MSA: multiple system atrophy; MSA-P: multiple system atrophy with predominant parkinsonism; UMSARS: Unified Multiple System Atrophy Rating Scale; PDSS-2: Parkinson’s disease sleep scale-2; ESS: Epworth sleepiness scale; RBDSQ: rapid eye movement sleep behavior disorder screening questionnaire; PD-SP: Parkinson’s disease related sleep problems; EDS: excessive daytime sleepiness; RBD: rapid eye movement sleep behavior disorder; LEDD: levodopa equivalent daily doses; FU: follow-up.

* Significant difference.

Table S2 The comparison of the score of PDSS-2 domain at baseline and 1- and 2-year follow-ups

| Time point | Disturbed sleep | p-value | p-value^#^ | Post hoc tests^#^ | Motor symptom at night | p-value | p-value^#^ | Post hoc tests^#^ | PD symptom at night | p-value | p-value^#^ | Post hoc tests^#^ |
| --- | --- | --- | --- | --- | --- | --- | --- | --- | --- | --- | --- | --- |
| MSA |  |  |  |  |  |  |  |  |  |  |  |  |
| Baseline | 6.85±4.05 | <0.001* | 0.015* | **a** | 1.57±1.83 | 0.836 | 0.664 | **-** | 1.18±2.01 | 0.014* | 0.060 | **-** |
| 1-year FU | 8.22±5.31 |  |  |  | 1.55±1.91 |  |  |  | 1.20±1.78 |  |  |  |
| 2-year FU | 8.72±5.61 |  |  |  | 1.76±2.01 |  |  |  | 1.88±2.22 |  |  |  |
| MSA-P |  |  |  |  |  |  |  |  |  |  |  |  |
| Baseline | 7.32±4.04 | 0.002* | 0.259 | - | 2.13±2.06 | 0.997 | 0.986 | **-** | 1.68±2.39 | 0.081 | 0.110 | **-** |
| 1-year FU | 8.84±5.40 |  |  |  | 2.11±2.26 |  |  |  | 1.69±2.11 |  |  |  |
| 2-year FU | 9.59±6.17 |  |  |  | 2.24±2.21 |  |  |  | 2.52±2.26 |  |  |  |
| MSA-C |  |  |  |  |  |  |  |  |  |  |  |  |
| Baseline | 6.40±4.03 | 0.002* | 0.023* | **a** | 1.04±1.40 | 0.628 | 0.636 | **-** | 0.70±1.43 | 0.177 | 0.290 | - |
| 1-year FU | 7.63±5.17 |  |  |  | 1.02±1.32 |  |  |  | 0.74±1.25 |  |  |  |
| 2-year FU | 7.82±4.86 |  |  |  | 1.25±1.66 |  |  |  | 1.20±1.98 |  |  |  |
| Male MSA |  |  |  |  |  |  |  |  |  |  |  |  |
| Baseline | 6.26±3.94 | 0.001* | 0.024* | **a** | 1.53±1.75 | 0.096 | 0.072 | **-** | 1.02±1.93 | 0.264 | 0.272 | - |
| 1-year FU | 7.69±5.12 |  |  |  | 1.18±1.72 |  |  |  | 0.94±1.68 |  |  |  |
| 2-year FU | 7.57±5.03 |  |  |  | 1.36±1.82 |  |  |  | 1.55±1.96 |  |  |  |
| Female MSA |  |  |  |  |  |  |  |  |  |  |  |  |
| Baseline | 7.47±4.09 | 0.001* | 0.115 | **-** | 1.62±1.93 | 0.234 | 0.640 | **-** | 1.35±2.09 | 0.035* | 0.074 | - |
| 1-year FU | 8.78±5.47 |  |  |  | 1.94±2.04 |  |  |  | 1.49±1.85 |  |  |  |
| 2-year FU | 9.83±5.96 |  |  |  | 2.13±2.14 |  |  |  | 2.20±2.42 |  |  |  |

MSA: multiple system atrophy; MSA-P: multiple system atrophy with predominant parkinsonism; MSA-C: multiple system atrophy with predominant cerebellar ataxia; PDSS-2: Parkinson’s disease sleep scale-2; FU: follow-up.

* Significant difference.

^#^Adjusting for age and LEDD.

Post hoc tests (Bonferroni correction):

Baseline vs 1-year follow-up: **a**, significant;

Baseline vs 2-year follow-up: **b**, significant;

1- vs 2-year follow-up: **c**, significant.

Table S3 Factors associated with score of PDSS-2, ESS, and RBDSQ in patients with MSA

| Variables | Score of PDSS-2 | | | Score of ESS | | | Score of RBDSQ | | |
| --- | --- | --- | --- | --- | --- | --- | --- | --- | --- |
|  | B | 95% CI | p-value | B | 95% CI | p-value | B | 95% CI | p-value |
| Sex (male=1) | -2.063 | -3.536 to -0.590 | 0.006* | 1.537 | 0.331 to 2.743 | 0.012* | -0.113 | -0.809 to 0.582 | 0.749 |
| Diagnosis subtype (MSA-P=1) | 3.146 | 1.626 to 4.666 | <0.001* | 3.066 | 1.829 to 4.303 | <0.001* | 0.095 | -0.619 to 0.808 | 0.795 |
| Age | 0.010 | -0.087 to 0.107 | 0.833 | 0.050 | -0.029 to 0.130 | 0.214 | -0.026 | -0.067 to 0.016 | 0.228 |
| UMSARS total score | 0.080 | 0.041 to 0.118 | <0.001* | 0.085 | 0.050 to 0.120 | <0.001* | 0.027 | 0.005 to 0.048 | 0.014* |
| OH | 0.722 | -0.322 to 1.766 | 0.175 | -0.230 | -0.973 to 0.513 | 0.545 | 0.514 | -0.043 to 1.070 | 0.070 |
| MoCA score | -0.084 | -0.259 to 0.092 | 0.352 | -0.282 | -0.436 to -0.129 | <0.001* | -0.004 | -0.087 to 0.079 | 0.929 |

MSA: multiple system atrophy; MSA-P: multiple system atrophy with predominant parkinsonism; UMSARS: Unified Multiple System Atrophy Rating Scale; PDSS-2: Parkinson’s disease sleep scale-2; ESS: Epworth sleepiness scale; RBDSQ: rapid eye movement sleep behavior disorder screening questionnaire; OH: orthostatic hypotension; MoCA, Montreal Cognitive Assessment.

* Significant difference.
